# Stress-induced symptom exacerbation: Stress increases voiding frequency, somatic sensitivity, and urinary bladder inflammation when combined with low concentration cyclophosphamide treatment in mice

**DOI:** 10.3389/fruro.2023.1079790

**Published:** 2023-03-22

**Authors:** Beatrice M. Girard, Susan E. Campbell, Margaret A. Vizzard

**Affiliations:** The Larner College of Medicine at The University of Vermont, Department of Neurological Sciences, Burlington, VT, United States

**Keywords:** flares, IC/BPS, psychological stress, bladder dysfunction, cystometry

## Abstract

Symptom exacerbation due to stress is prevalent in many disease states, including functional disorders of the urinary bladder (e.g., overactive bladder (OAB), interstitial cystitis/bladder pain syndrome (IC/BPS)); however, the mechanisms underlying the effects of stress on micturition reflex function are unclear. In this study we designed and evaluated a stress-induced symptom exacerbation (SISE) mouse model that demonstrates increased urinary frequency and somatic (pelvic and hindpaw) sensitivity. Cyclophosphamide (CYP) (35 mg/kg; i.p., every 48 hours for a total of 4 doses) or 7 days of repeated variate stress (RVS) did not alter urinary bladder function or somatic sensitivity; however, both CYP alone and RVS alone significantly (p ≤ 0.01) decreased weight gain and increased serum corticosterone. CYP treatment when combined with RVS for 7 days (CYP+RVS) significantly (p ≤ 0.01) increased serum corticosterone, urinary frequency and somatic sensitivity and decreased weight gain. CYP+RVS exposure in mice significantly (p ≤ 0.01) increased (2.6-fold) voiding frequency as we determined using conscious, open-outlet cystometry. CYP+RVS significantly (p ≤ 0.05) increased baseline, threshold, and peak micturition pressures. We also evaluated the expression of NGF, BDNF, CXC chemokines and IL-6 in urinary bladder in CYP alone, RVS alone and CYP+RVS mouse cohorts. Although all treatments or exposures increased urinary bladder NGF, BDNF, CXC and IL-6 content, CYP+RVS produced the largest increase in all inflammatory mediators examined. These results demonstrated that CYP alone or RVS alone creates a change in the inflammatory environment of the urinary bladder but does not result in a change in bladder function or somatic sensitivity until CYP is combined with RVS (CYP+RVS). The SISE model of CYP+RVS will be useful to develop testable hypotheses addressing underlying mechanisms where psychological stress exacerbates symptoms in functional bladder disorders leading to identification of targets and potential treatments.

## 1 Introduction

Animal models are used to mirror interstitial cystitis/bladder pain syndrome (IC/BPS) pathophysiology to increase our understanding of the underlying mechanisms and identify potential targets for treatment interventions. Patients with lower urinary tract (LUT) disorders report worse symptoms during stress (1, 2). Stress may contribute to the exacerbation and likely the development of LUT disorders including IC/BPS and overactive bladder (OAB) (3–6). A majority of IC/BPS patients report exacerbation of symptoms by clinical stress (6–8), and experimental stress increases bladder pain and urgency (9). Individuals with urologic chronic pelvic pain syndrome (UCPPS) exhibit greater lifetime stress and widespread pain symptoms and less resiliency and coping mechanisms (10).

Animal models of stress demonstrate symptoms of bladder dysfunction (e.g., increased micturition frequency, pain) as well as anxiety-like behavior (3) that may be due, in part, to dysregulation of the hypothalamic-pituitary-adrenal (HPA) axis. Individuals with bladder dysfunction disorders may have abnormalities in the HPA axis, and stress may contribute to the increase in bladder symptoms (1, 2). Although stress may exacerbate LUT symptoms, the pathophysiology underlying the development and progression of stress on urinary frequency and pelvic pain remains unknown.

Symptom exacerbation, often referred to as flares, among patients with IC/BPS is well recognized and attributed to multiple triggers, including infection, diet, physical activities, sexual activities, allergies, and stress (6, 11–13). The proportion (15-95%) of IC/BPS patients with flares varies widely (6, 11–13). Although flares vary in terms of duration, frequency, intensity, and type of symptoms, flares are bothersome, disruptive, and associated with increased pelvic pain and urologic symptoms (6, 11–13). Few studies have addressed symptom flare in IC/BPS patients and basic research investigations of flares are hampered by the lack of animal models that mimic symptom flare upon which one can then develop testable hypotheses (6, 11–13).

In this study, we describe the initial characterization a mouse model where administration of cyclophosphamide (CYP) (35 mg/kg; i.p., every 48 hours for a total of 4 doses) when combined with 7 days of stressor presentation results in increased voiding frequency and increased somatic sensitivity. Neither the administration of CYP or the 7 days of repeated variate stress (RVS) (14–17) alone increased voiding frequency or somatic sensitivity in C57Bl/6 mice. We refer to this mouse model as a stress-induced symptom exacerbation (SISE) mouse model. In this paper, we describe our characterization of the SISE model including, bladder function with conscious, open outlet continuous cystometry and somatic sensitivity with von Frey filament testing. We have also examined corticosterone expression, and expression of inflammatory mediators including neurotrophic factor (NGF, BDNF) (18–20), chemokine (CXC) and interleukin-6 (IL-6) (18, 21–34) expression in the urinary bladder. These initial results demonstrated that CYP alone, or RVS alone creates a change in the inflammatory environment of the urinary bladder and a systemic increase in corticosterone expression but does not result in a change in bladder function or somatic sensitivity until CYP is combined with RVS (CYP+RVS).

## 2 Methods

### 2.1 Animals

**Figure 1:**
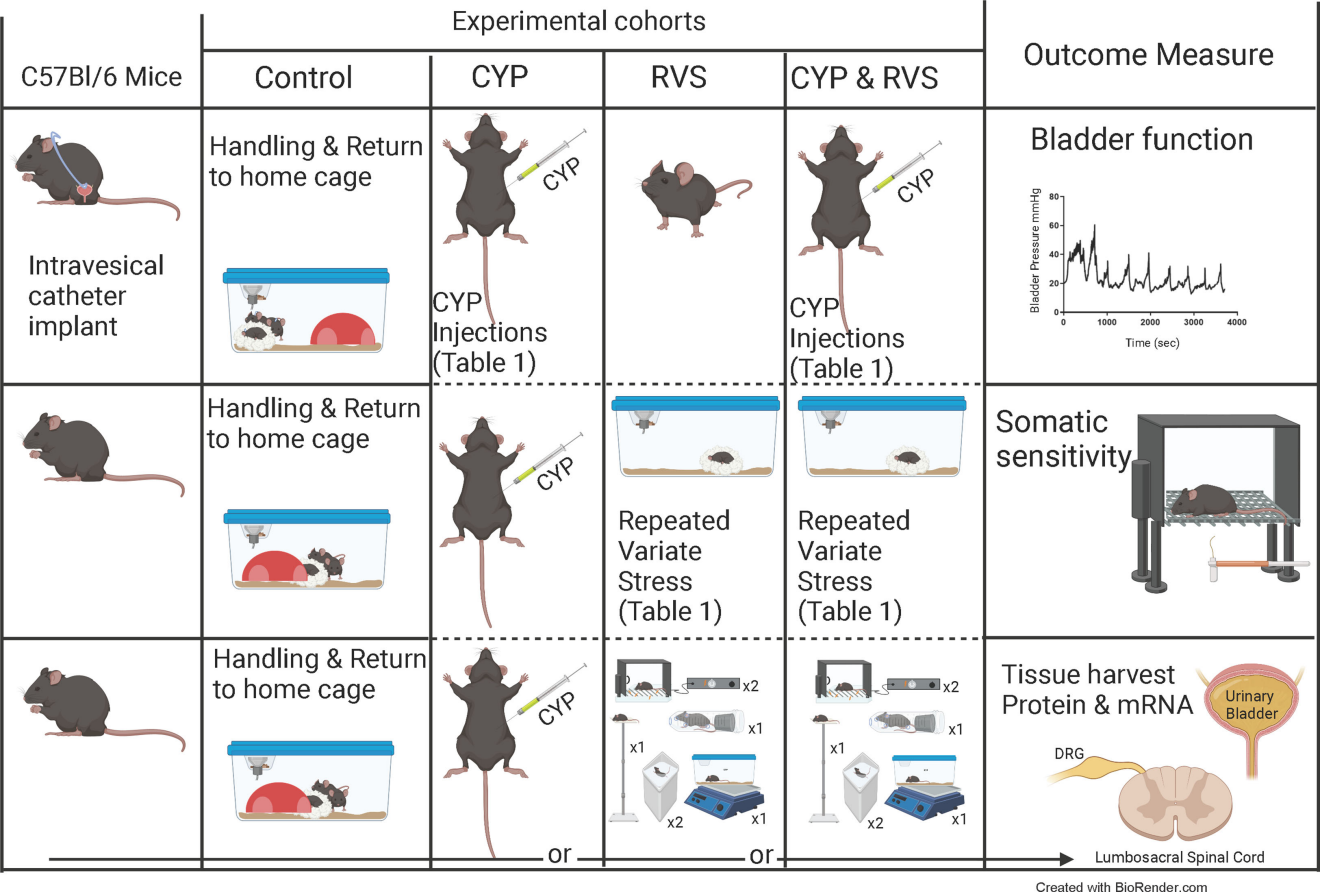
Experimental cohorts and experimental outcomes. Overview of studies using control (C57Bl/6) mice (handled and returned to home cage), mice treated with cyclophosphamide (CYP) alone, mice exposed to repeated variate stress (RVS) alone, or mice exposed to both CYP + RVS. Primary outcome measures measured in this study: urinary bladder function and referred somatic sensitivity of the pelvic region and hindpaw. Tissues were also harvested for current and future studies. The RVS protocol (7 day) consisted of 5 different stressors with a novel stressor delivered each day (see methods for additional details including duration of stressors) (14, 37–39). Swim and foot shock stressors are repeated on the last two days of RVS. sec, seconds; DRG, dorsal root ganglia.

Female C57BL/6 wildtype (WT) mice used in this study were bred locally at the Larner College of Medicine at the University of Vermont. To maintain diversity in this colony, mice (C57BL/6J) were also purchased from The Jackson Laboratory and incorporated into breeding. The litters were average in size (6-8 pups), weight, and activity (feeding, drinking, behaviors) with consistency in the overall proportion of male and female mice. The UVM Institutional Animal Care and Use Committee approved all experimental protocols involving animal usage (IACUC #X9-020). Animal Care was under the supervision of the UVM Office of Animal Care Management in accordance with the Association for Assessment and Accreditation of Laboratory Animal Care (AAALAC) and National Institutes of Health (NIH) guidelines. We used female mice because of the female predominance of IC/BPS (24, 35, 36). Estrous cycle status was not determined for mice in these studies. All efforts were made to minimize the potential for animal pain, stress, or distress. Separate groups of littermate WT were used in the following experiments. Mice were randomly assigned to control, CYP, RVS or CYP+RVS cohorts (N = 8) after weaning and weighed daily (**Figure 1**). Cohorts consisted of mice from 3-5 litters.

#### 2.1.1 CYP-induced cystitis

Mice (N = 8, per treatment group) received cyclophosphamide (CYP) (35 mg/kg every 48 hours for a total of 4 injections) intraperitoneally (i.p.) to create a mouse model that did not exhibit changes in bladder or somatic function in the absence of RVS (**Figure 1**) (**Table 1**). CYP is metabolized to acrolein, an irritant expelled in the urine (40–42). Injections were performed in mice anesthetized with isoflurane (3%). The control group received no CYP treatment. CYP alone and CYP+RVS groups received the same dose and frequency of administration described here.

**Table 1:** Timeline of dosing schedule and stressor exposure for the CYP, RVS or CYP+RVS mice groups.

Timeline	D1	D2	D3	D4	D5	D6	D7	D8	D9
CYP injection	CYP		CYP		CYP		CYP		Experimental Outcome: Somatic sensitivity Bladder function Tissue H
RVS	Stressor (S)	S	S	S	S	S	S		
RVS + CYP	CYP + S	S	CYP + S	S	CYP + S	S	CYP + S		

On days 8-9, neither CYP nor exposure to stress occurred as mice recovered postoperatively from bladder catheter implant surgery before determination of experimental outcomes.

#### 2.1.2 Repeated variate stress

Mice assigned to the RVS group (hereafter RVS mice) or CYP+RVS (hereafter CYP+RVS mice) group were exposed to 7 days of stressors with a single stressor being presented on each day between 9 am-12 pm as described previously (14, 15, 37, 38) (**Figure 1**). Control mice (no stress or CYP) were handled daily and remained in home cages in the animal facility following weighing. Oscillation stress: Mice were placed inside a plastic chamber 25 × 16 ×13 cm (L ×W × H), that was secured to a clinical rotator (Fisher Scientific, Morris Plains, NJ), and oscillated at low to medium speed for 30 minutes (min). Forced swim: Mice were placed in a cylindrical container 29 × 37 cm (D × H) that was filled with room temperature water to a depth that prevented the tail from touching the bottom of the container. After 5 min of monitored swimming, mice were placed in a holding chamber for 30 min prior to being returned to their home cage. Electrical foot shock: Mice were placed inside a Plexiglass conditioning chamber (Med Associates, St. Albans, VT) 30 × 25 × 35 cm (L × W × H). After a 5 min acclimation period, two 0.2 mA, 5 second (s) scrambled foot shocks were delivered through the grid floor with a 1 min inter-trial interval. Restraint: Mice were placed in a cylindrical restraining device 30 × 115 mm (D × L) for 60 min. Pedestal: Mice were placed on an elevated (60 cm) platform 20 × 20 cm (L × W) for 30 min. Forced swim and electrical foot shock were the 2 stressors repeated on days (D) 6 and D7 (**Table 1**; **Figure 1**).

#### 2.1.3 CYP+RVS

Mice (N = 8, per treatment group) received cyclophosphamide (CYP) (35 mg/kg every 48 hours for a total of 4 injections) (Day(D) 1, D3, D5 and D7) (**Figure 1**, **Table 1**) intraperitoneally (i.p.) to create a mouse model that did not exhibit changes in bladder or somatic function in the absence of RVS. The CYP dosing schedule (**Table 1**) was combined with daily (D1-D7) exposure to various stressors (**Figure 1**) described above. In pilot studies, we evaluated different doses of CYP (15, 25, 30, 35 mg/kg) at the same dosing frequency that when combined with RVS would result in increased voiding frequency. Neither the frequency of CYP dosing nor the duration of the RVS protocol was altered. Intraperitoneal injection of CYP (35 mg/kg; every 48 hours for a total of 4 doses) combined with 7-day RVS in female mice resulted in the most consistent increases in voiding frequency.

### 2.2 Conscious, open outlet, continuous fill cystometry

On D7, mice in all treatment groups (N = 8 for each: control, CYP, RVS, CYP+RVS) were anesthetized with isoflurane (3-4%) in 100% O_2_, a lower midline abdominal incision was made, and polyethylene tubing (PE-10, Clay Adams, Parsippany, New Jersey) was inserted into the bladder dome and secured with a nylon purse-string suture (6-zero) (14, 43–45). The end of the PE tubing was heat flared, but the catheter did not extend into the bladder body or neck, and it was not associated with inflammation or altered cystometric function (14, 43–45). The distal end of the tubing was sealed, tunneled subcutaneously, and externalized at the back of the neck (14, 43–45). Abdominal and neck incisions were closed with nylon sutures (6–0). Mice received postoperative analgesics (subcutaneous carprofen, 5.0 mg/kg, once a day for two days) and recovered from survival surgery for 48 hr before performing cystometry. Mice were returned to home cages and no stressor or CYP administration occurred for the remaining 2 days of the protocol (**Table 1**).

For cystometry, a conscious, unrestrained mouse was placed in a Plexiglas cage with a wire bottom. Prior to the start of recording, the bladder was emptied, and the catheter was connected *via* a T-tube to a pressure transducer (Grass Model PT300, West Warwick, RI) and microinjection pump (Harvard Apparatus 22, South Natick, MA). A Small Animal Cystometry Lab Station (MED Associates, Fairfax, VT) was used for urodynamic measurements (14, 43–45). Saline solution was infused at room temperature into the bladder at a rate of 25 µl/min to elicit repetitive bladder contractions. At least six reproducible micturition cycles were recorded after the initial stabilization period of 25–30 min (14, 43–45). The following cystometric parameters were recorded in each animal: baseline pressure (pressure at the beginning of the bladder filling), threshold pressure (bladder pressure immediately prior to micturition), peak micturition pressure, intercontraction interval (ICI; time between micturition events), infused volume (IV), void volume (VV) (14, 43–45). Mice in these studies had residual volume of less than 10 μl. At the conclusion of the experiment, the mouse was euthanized (5% isoflurane plus thoracotomy). Experiments were conducted at similar times of the day (9 am -12 pm) to avoid the possibility that circadian variations were responsible for changes in bladder capacity measurements (14, 43–45). An individual blinded to treatment or group analyzed the cystometric data; groups were decoded after data analysis.

### 2.3 Exclusion criteria

Mice were removed from the study when adverse events occurred that included a significant postoperative event, lethargy, pain, or distress not relieved by our IACUC-approved regimen of postoperative analgesics (14, 43–45). In addition, behavioral movements such as grooming, standing, walking, and defecation rendered bladder pressure recordings during these events unusable. Approximately 10% of the total number of mice dedicated to these studies were removed from the study.

### 2.4 Mechanical sensitivity testing

Referred (secondary) hyperalgesia was measured by testing the frequency of withdrawal responses to the application of calibrated von Frey monofilaments to the abdominal (14, 43–45) region overlying the urinary bladder + suprapubic region and the hindpaw. Mice were returned to home cages, weighed daily, but no stressor or CYP administration occurred for the remaining 2 days of the protocol (**Table 1**) before somatic sensitivity testing. Separate groups (N = 10 each) of mice were evaluated: control, CYP, RVS, CYP+RVS. Mechanical sensitivity assessment was performed using von Frey monofilaments (Stoelting, Wood Dale, IL) with forces of 0.1–4 g applied to the pelvic region (14, 43–45). Mechanical sensitivity was then evaluated in the hindpaw in an identical manner as previously described (14, 43–45). Testing of the plantar region of the hindpaw and lower abdominal area was performed by perpendicular application of von Frey filaments until the hair bent slightly. All mice were first habituated in a clear acrylic testing chamber for 20 min/day for 4 days prior to the initiation of the CYP, RVS or CYP+RVS treatment or handling (control) with a fan to generate ambient noise. On day of testing (D9) (**Table 1**), the mice were placed in the acrylic testing chamber on top of a metal mesh floor (IITC Life Science Inc., Woodland Hills, CA) and habituated again for 10 min before the application of von Frey filaments in an up-down method for 1–3 s with a minimum interstimulus interval of 2 min (14, 43–45). The following behaviors were considered positive responses to pelvic region stimulation: sharp retraction of the abdomen, jumping, or immediate licking or scratching of the pelvic area (14, 43–45). A positive response to hindpaw stimulation was sharp withdrawal of the paw or licking of the tested hindpaw (14, 43–46). All somatic testing was performed in a blinded manner. The groups were decoded after data analysis. Separate cohorts of mice were used for cystometry and somatic sensitivity testing to avoid any potential confounding events of an abdominal incision.

### 2.5 Euthanasia and tissue harvest

Female mice were deeply anesthetized with isoflurane (5%) in 100% O_2_, blood from the abdominal aorta was collected (see below), the urinary bladder dissected and then mice were euthanized *via* thoracotomy. The urinary bladder was quickly snap-frozen on dry ice prior to processing (47). In addition, urinary bladders from some mice were used to determine if the lower concentration (35 mg/kg) of CYP produced inflammatory changes in the urinary bladder. Urinary bladders were harvested, cut along the midline, opened and pinned to Sylgard-coated plates and photographed. The pinned bladders were then prepared for tissue sectioning (15 µm), H+E staining and brightfield microscopy (Olympus BX40) as previously described (20, 41).

### 2.6 Measurement of urinary bladder NGF, BDNF, CXCL9, CXCL10, IL-6 expression and serum corticosterone

We determined serum corticosterone from control, CYP, RVS and CYP+RVS mice (N = 8 each) using ELISAs (ADI‐900‐097, Enzo Life Sciences, Farmingdale, NY) (14). At time of euthanasia on D9 (**Table 1**), blood from the abdominal aorta was collected in serum tubes between 9 am-12 pm and allowed to coagulate at room temperature for 1 hr. Samples were centrifuged in an Eppendorf 5415R centrifuge at 10,000 rpm for 10 min. Serum was aliquoted and frozen at −20 °C until analysis. We determined NGF and BDNF, IL-6 and CXC (CXCL9, CXCL10) protein content in the urinary bladder of control, CYP, RVS and CYP+RVS treatment groups (N=8) using ELISAs (Promega, Madison, WI and R&D Systems, Minneapolis, MN) as previously described (18, 19, 31, 32, 48). According to the manufacturer, the NGF E-max or BDNF E-max immunoassay systems (Promega, Madison, WI) demonstrate very low cross-reactivity with structurally related growth factors. We have recently (20) determined urinary bladder NGF expression under various experimental conditions and compared results obtained with Promega and Biosensis (BioSensis, Thebarton SA, Australia) kits and found no statistical differences between results. According to the manufacturer, no significant cross-reactivity or interference is observed with other cytokines or chemokines with any Quantikine M immunoassay kit (R&D Systems, Minneapolis, MN) used in the present study for IL-6 or CXC detection. The standards provided with these systems generated linear standard curves (R*^2^* = 0.996–0.998, p ≤ 0.001). Absorbance values of standards and samples were corrected by subtraction of the background value (absorbance due to nonspecific binding). No samples were diluted, and all samples had absorbance values on the linear portion of the standard curve. Curve fitting of standards and evaluation of NGF, BDNF, CXC, IL-6 or corticosterone content of samples were performed using a least-squares fit.

### 2.7 Statistical analyses

All values represent mean ± SEM. Normality of data was verified using the D’Agostino-Pearson normality test (GraphPad Prism). Comparisons among experimental groups were made using analysis of variance (ANOVA), repeated measures ANOVA, paired or unpaired t test where appropriate. When F-test statistic exceeded the critical value at α = 0.05, the Sidak’s multiple comparisons test was used to compare group means.

## 3 Results

### 3.1 Body weight decreased, and serum corticosterone increased in mice exposed to CYP, RVS, or CYP+RVS

**Figure 2:**
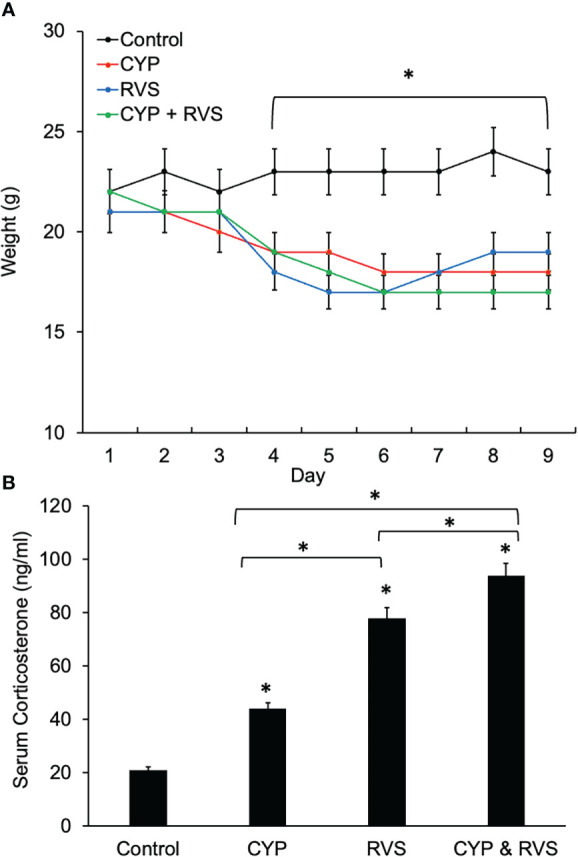
Control, CYP, RVS or CYP+RVS effects on serum corticosterone, and body weight **(A)** Changes in body weight of mice over 9 days for mice in control, CYP, RVS or CYP+RVS cohorts. Beginning on day 4 and lasting for the full 9 day duration of study, mice in CYP, RVS, or CYP+RVS cohorts exhibited significant weight gain attenuation compared with control mice. Body weights were significantly (p ≤ 0.01) decreased in CYP, RVS or CYP+RVS mice groups from days 4–9. **(B)** Serum corticosterone significantly (p ≤ 0.01) increased in CYP, RVS or CYP+RVS mice groups exposure measured on day 9 of the study duration. Mice in the CYP+RVS group exhibited the greatest (4.5-fold) increase in serum corticosterone compared to CYP (2.1-fold) or RVS (3.7-fold) alone groups. B: Samples size are n = 8. Values are means ± SEM. *p ≤ 0.01.

CYP, RVS or CYP+RVS significantly (p ≤ 0.01) decreased body weight in mice by day 4 of the CYP alone, RVS alone or combined CYP+RVS protocols (**Figure 2A**). Significant (p ≤ 0.01) weight reductions were maintained throughout the study (day 9) of the protocols (**Figure 2A**). CYP, RVS or CYP+RVS RVS significantly (p ≤ 0.01) increased serum corticosterone measured on day 9 compared to control mice (handling only) (**Figure 2B**); however, the magnitude of increase varied with treatment. Combined CYP+RVS treatment resulted in the greatest increase in serum corticosterone (4.5-fold) followed by increases resulting from RVS alone (3.7-fold) or CYP alone (2.1-fold) (**Figure 2B**).

### 3.2 Urothelial petechial hemorrhages and urothelial erosion in mice treated with CYP

**Figure 3:**
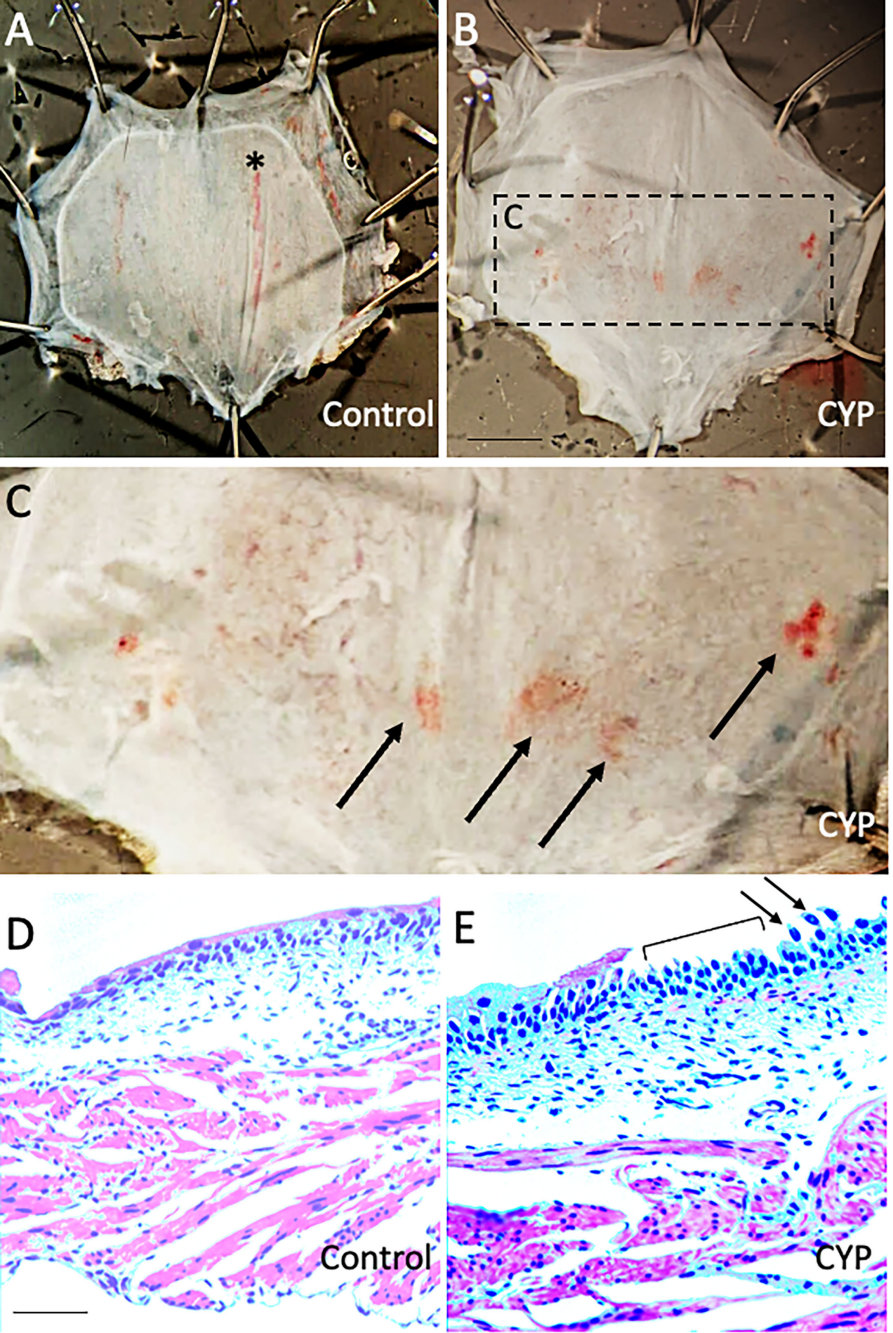
Urinary bladders from mice treated with low concentration CYP (35 mg/kg, i.p., every 48 hours for 7 days) exhibit urothelial petechial hemorrhages and erosion. **(A, B)** Gross images of urinary bladder from control **(A)** and CYP-treated mice **(B)**. Bladders from CYP-treated mice **(B)** exhibit petechial hemorrhages on the urothelial surface compared to control mice **(A)**. The rectangular region in **B** is enlarged in **C** to highlight regions of petechial hemorrhage (arrows). In control bladder **(A)**, there is a vertically extending, large blood vessel (*). **(D, E)** H+E staining in urinary bladder sections (10 µm) from control **(D)** and CYP-treated mice **(E)**. In CYP-treated mice, urinary bladder sections exhibit regions of urothelium erosion (**E**, bracket) and urothelial cell sloughing (**E**, arrows). In control mice, the urothelium was intact **(D)**. Calibration bar in **B** represents: 1.5 mm in **A-B**, 0.6 mm in **C** and 100 µm in **D, E**.

We previously demonstrated inflammatory changes in the urinary bladder in rodents treated with intraperitoneal CYP when used at a concentration of 75 mg/kg with a frequency of treatment of every 48-72 hours for 8-10 days (41, 49). In the current study, we treated mice with intraperitoneal CYP at a reduced concentration (35 mg/kg) administered every 48 hours for 7 days. Urinary bladders harvested from mice treated with CYP showed evidence of petechial hemorrhages on the urothelial surface (**Figures 3A-C**) as well as areas of urothelial erosion and urothelial cell sloughing in tissue sections compared to control mice. Regions of urothelial erosion and sloughing were patchy with intact regions of urothelium also being observed (**Figure 3E**). In contrast, urinary bladder from control mice exhibited intact urothelium throughout the full extent of tissue sections (**Figure 3D**).

### 3.3 Urinary bladder NGF and BDNF expression increased in mice exposed to CYP, RVS or CYP+RVS

**Figure 4:**
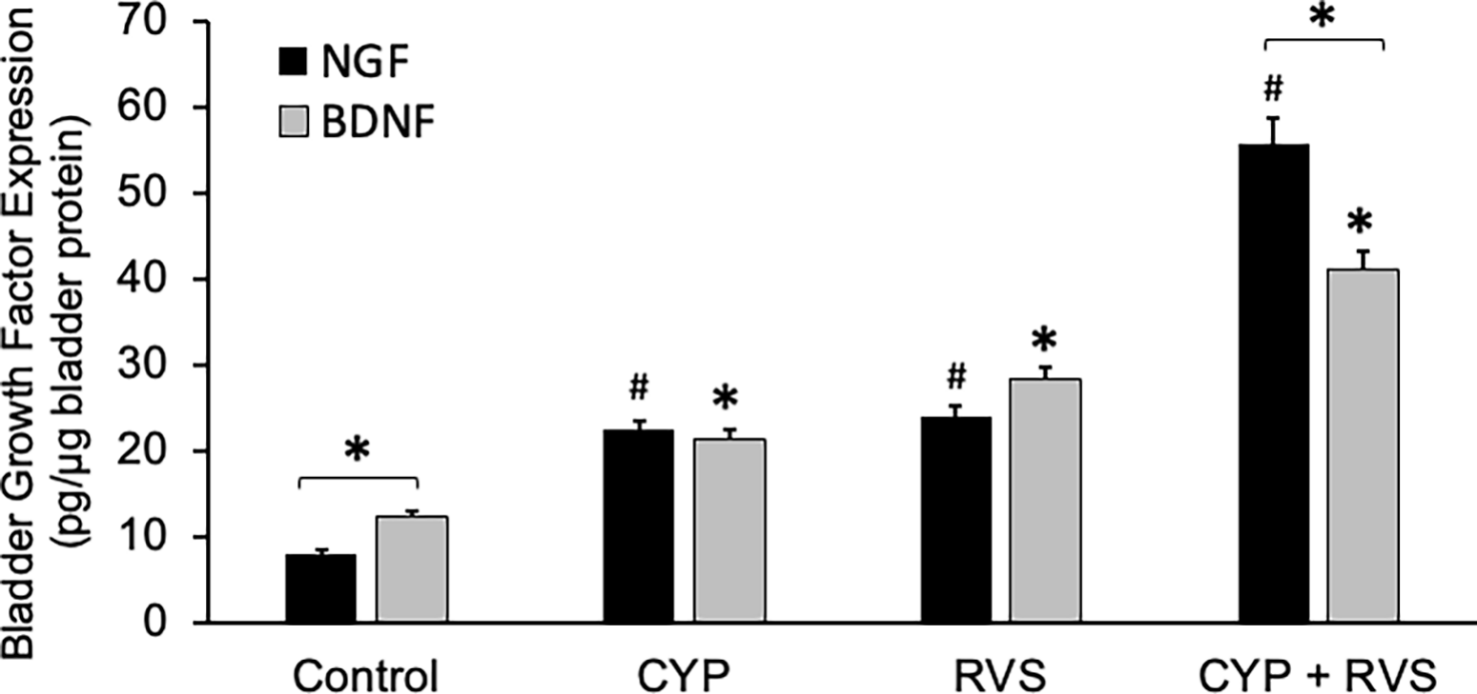
Increased Expression of NGF and BDNF in Urinary Bladder with CYP, RVS or CYP+RVS. Basal expression of urinary bladder BDNF was significantly (p ≤ 0.01) greater than NGF expression in control mice. CYP, RVS and CYP+RVS significantly (p ≤ 0.01) increased both NGF and BDNF urinary bladder expression. CYP+RVS induced the greatest increase in NGF and BDNF urinary bladder expression compared to CYP or RVS alone. Urinary bladder NGF expression was significantly (p ≤ 0.01) greater than BDNF urinary bladder expression in mice exposed to CYP+RVS. Samples size are n = 8. Values are means ± SEM. * or #, p ≤ 0.01. Bracket and * indicates comparison between NGF and BDNF expression.

CYP alone, RVS alone or combined CYP+RVS significantly (p ≤ 0.01) increased whole urinary bladder NGF in mice measured on day 7 of the RVS paradigm (**Figure 4**). Although urinary bladder NGF increased expression with CYP alone (2.8-fold) and RVS alone (3.1-fold) protocols, the greatest increase (8.1-fold) in urinary bladder NGF expression was demonstrated following the combined CYP+RVS protocol (**Figure 4**). A similar observation was made with urinary bladder BDNF expression where combined CYP+RVS produced the greatest increase (3.3-fold) in expression compared to CYP alone (1.8-fold) or RVS alone (2.3-fold) protocols (**Figure 4**). Baseline BDNF urinary bladder expression was greater than NGF expression, but urinary bladder NGF expression was significantly (p ≤ 0.01) greater than BDNF following CYP+RVS (**Figure 4**). No differences between urinary bladder NGF or BDNF expression were observed with CYP alone or RVS alone protocols (**Figure 4**).

### 3.4 Urinary bladder CXC and IL-6 expression increased in mice exposed to CYP, RVS, or CYP+RVS

**Figure 5:**
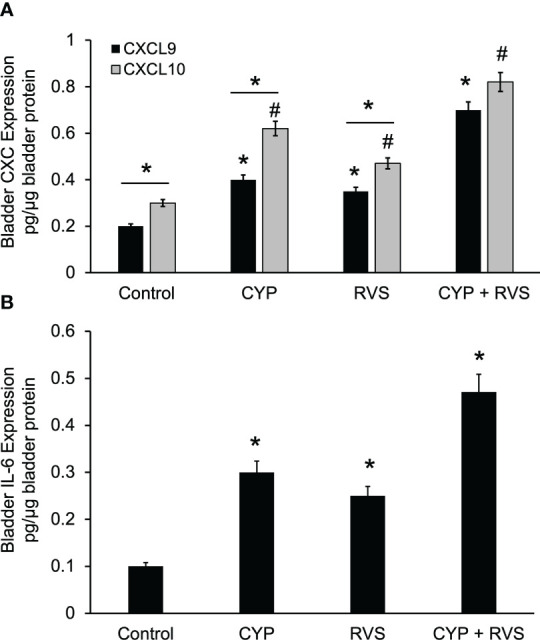
Increased Expression of CXC Chemokines and IL-6 in Urinary Bladder with CYP, RVS or CYP+RVS. **(A)** CYP alone, RVS alone or combined CYP+RVS significantly (p ≤ 0.01) increased whole urinary bladder CXCL9 (*, p ≤ 0.01)) and CXCL10 (#, p ≤ 0.01) expression in mice. Although urinary bladder CXCL9 and CXCL10 increased expression with CYP alone (2.0-2.1-fold) and RVS alone (1.5-1.8-fold) protocols, the greatest increases (2.7-3.5-fold) in expression were demonstrated with combined CYP+RVS **(A)**. Baseline (control), CYP alone and RVS alone CXCL10 urinary bladder expression was significantly (line with *, p ≤ 0.01) greater than CXCL-9 expression; however, no differences were observed between CXCL9 and CXCL10 expression with combined CYP+RVS treatment **(A)**. **(B)** Urinary bladder IL-6 expression was significantly (*, p ≤ 0.01) increased with CYP alone, RVS alone and CYP+RVS. The largest increase in IL-6 bladder expression was demonstrated with the combined CYP+RVS protocol compared to CYP alone (3.0-fold) or RVS alone (2.5-fold) treatment protocols **(B)**. Samples size are n = 8. Values are means ± SEM. * or #, p ≤ 0.01. Line and * indicates comparison between CXCL9 and CXCL10 expression.

CYP alone, RVS alone or combined CYP+RVS significantly (p ≤ 0.01) increased whole urinary bladder CXCL9 and CXCL10 expression (**Figure 5A**). Although urinary bladder CXCL9 and CXCL10 increased expression with CYP alone (2.0-2.1-fold) and RVS alone (1.5-1.8-fold) protocols, the greatest increases (2.7-3.5-fold) in expression were demonstrated with the combined CYP+RVS protocol (**Figure 5A**). Baseline (control), CYP alone and RVS alone CXCL10 urinary bladder expression was significantly (p ≤ 0.01) greater than CXCL-9 expression; however, no differences were observed between CXCL9 and CXCL10 expression with combined CYP+RVS treatment (**Figure 5A**). Urinary bladder IL-6 expression was greatest with the combined CYP+RVS protocol compared to CYP alone (3.0-fold) or RVS alone (2.5-fold) treatment protocols (**Figure 5B**).

### 3.5 CYP+RVS increases voiding frequency and decreases functional bladder capacity in female mice

**Figure 6:**
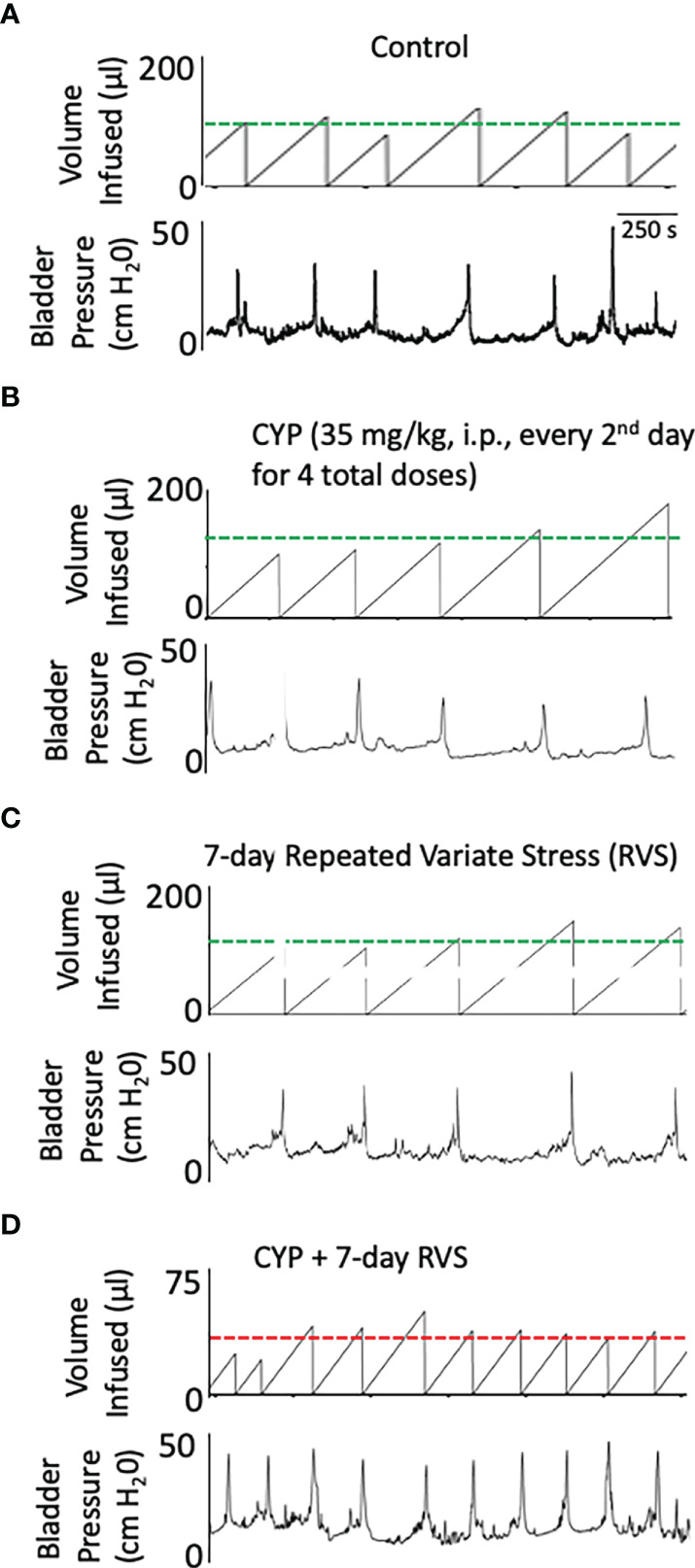
Representative bladder function recordings showing volume infused and bladder pressure recorded during conscious, open outlet cystometry. Bladder function recordings from control **(A)**, CYP (35 mg/kg, i.p., every 48 hours for 4 total doses), **(B)** RVS (7 day), **(C)** or CYP+RVS (7 day). **(A)** Control mice (handling and returned to home cage) infused intravesically with saline (0.9%) exhibited an infused volume (IV) necessary to elicit a micturition event (i.e., functional bladder capacity) that is like CYP **(B)** and RVS alone **(C)**. **(D)**. CYP+RVS mice exhibited a reduced infused volume (IV) required to elicit a micturition that results in increased voiding frequency compared to control, CYP or RVS mice. Timeline represents 250 s from the recorded cystometrogram.

**Figure 7:**
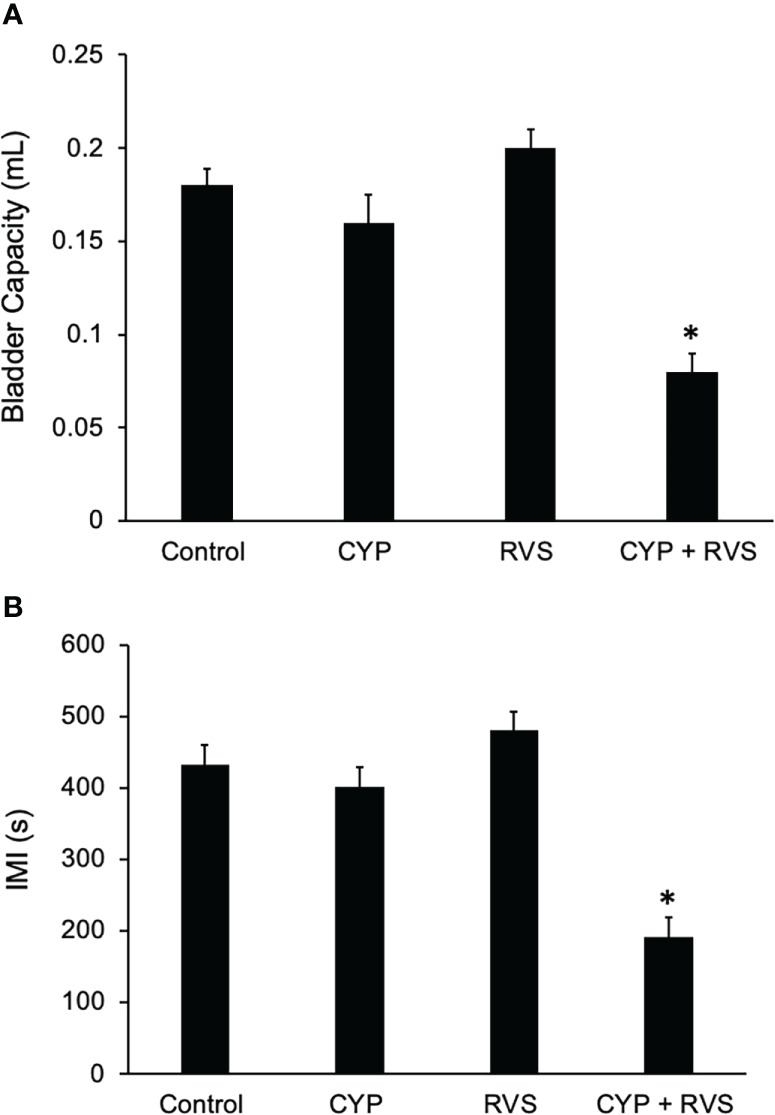
Summary histograms of functional bladder capacity **(A)** (mL) and intercontraction interval **(B)** (ICI) measured from bladder function testing in control, CYP, RVS and CYP+RVS mice. CYP+RVS mice exhibited significantly (*, p ≤ 0.01) reduced IV **(A)** and ICI **(B)** compared to control (handled), CYP alone and RVS alone mice. Values are means ± SEM. Samples size are n = 8; *, p ≤ 0.01 versus control. Seconds, s; milliliter, mL.

In this study, we selected a concentration of CYP (35 mg/kg, i.p.) and frequency of administration that when given alone, in the absence of RVS, did not affect voiding frequency (**Figure 6B**) compared to control (**Figure 6A**). In C57Bl/6 female mice exposed to 7-day RVS, no changes in voiding frequency were observed compared to control (handled) (**Figure 6C**). CYP when combined with 7-day RVS, significantly increased voiding frequency (**Figure 6D**). Summary data demonstrate that CYP+RVS significantly (p ≤ 0.01) decreased functional bladder capacity (2.4-fold *vs*. control) (infused volume that elicits a void event) (**Figure 7A**) and significantly (p ≤ 0.01) reduced the intermicturition interval (IMI) (2.4-fold *vs*. control) (increased voiding frequency) (**Figure 7B**). The magnitude of change in bladder capacity (2.2-2.5-fold) and IMI (2.1-2.6-fold) in the CYP+RVS cohort was similar when compared to each group (control, CYP, or RVS). No changes were observed in CYP alone or RVS alone cohorts when compared to control (**Figures 7A, B**).

### 3.6 CYP+RVS exposure increases baseline, threshold, and peak micturition pressures

C57Bl/6 female mice exposed to CYP+RVS significantly (p ≤ 0.05) increased baseline, threshold, and peak micturition pressures (**Figure 2**) (**Table 2**). No changes in baseline, threshold or peak micturition pressures were observed in CYP alone or RVS alone cohorts compared to control (**Figure 2**, **Table 2**).

**Table 2:** Bladder pressures (baseline, threshold, peak micturition) recorded during conscious cystometry in control, CYP, RVS or CYP+RVS mice.

Pressure (cm H_2_0)	Control	CYP	RVS	CYP+RVS
Baseline	10.5 ± 2.3	12.1 ± 2.1	15.4 ± 2.7	17.2 ± 3.2#
Threshold	13.3 ± 1.7	15.7 ± 2.4	18.1 ± 2.5	20.3 ± 3.4*
Micturition	38.4 ± 4.3	37.3 ± 2.8	40.9 ± 3.5	46.8 ± 3.6#

CYP+RVS mice exhibited significant increases in baseline, threshold and peak micturition pressure compared to control mice. Values are means ± SEM. Samples size are n = 8; *p ≤ 0.01; #p ≤ 0.05.

### 3.7 CYP+RVS increases pelvic and hindpaw somatic sensitivity in female mice

**Figure 8:**
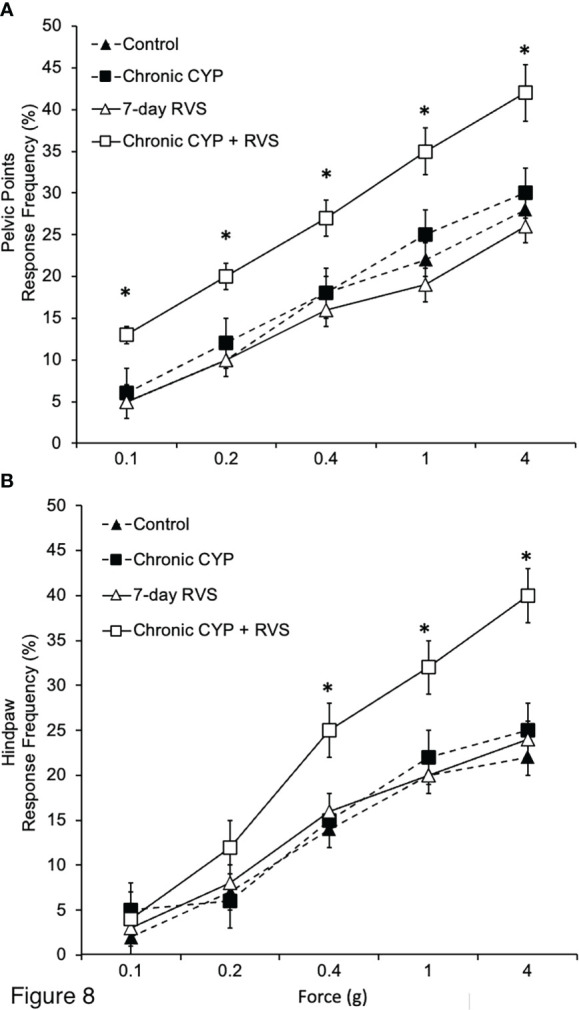
Effects of CYP, RVS or CYP+RVS on pelvic and hindpaw region sensitivity as determined using calibrated von Frey filaments. **(A)** Pelvic stimulation was applied to the lower abdominal area, including the suprapubic region, overlying the urinary bladder. CYP+RVS mice (n = 10) exhibited significantly (*, p ≤ 0.01) increased pelvic response frequency with all von Frey filaments (0.1–4 g) tested compared to control mice, CYP or RVS alone (n = 10). **(B)** CYP+RVS mice (n = 10) exhibited significantly (*, p ≤ 0.01) increased hindpaw response frequency with all von Frey filaments (0.1–4 g) tested compared to control mice, CYP or RVS alone (n = 10). All somatic testing was performed blinded. Values are means ± SEM. Samples size are n = 10; *p ≤ 0.01.

Female mice exposed to CYP+RVS exhibited significantly (p ≤ 0.01) decreased somatic sensitivity in the pelvic region (**Figure 8A**) with all monofilament forces evaluated (0.1 to 4 g). Female mice exposed to CYP+RVS exhibited significantly (p ≤ 0.01) decreased somatic sensitivity to hindpaw stimulation with monofilaments of greater force (0.4-4 g). Control, CYP alone or RVS alone mouse cohorts exhibited similar somatic sensitivity in the pelvic region (**Figure 8A**) and hindpaw (**Figure 8B**) with all monofilament forces evaluated (0.1 to 4 g).

## 4 Discussion

Interstitial cystitis/bladder pain syndrome (IC/BPS) is an unpleasant sensation (pain, pressure, discomfort) perceived to be related to the urinary bladder, associated with LUT symptoms of more than six weeks duration, in the absence of infection or other identifiable causes (50). Symptom exacerbation (flare) is common with multiple, perceived triggers including stress. The impact of BPS/IC on quality of life is enormous (51) and flares are bothersome and disruptive (6, 11–13). Basic research investigations of flares are hampered by the lack of animal models (11–13). Effective treatments and a greater understanding of the contribution of stress to BPS/IC are needed.

A majority (78.4%) of participants enrolled in a Multidisciplinary Approach to the Study of Chronic Pelvic Pain (MAPP) study (6) that evaluated management and patient-reported flares in IC/BPS indicated that stress is a perceived trigger for their flares. Other contributing causes include specific foods, physical activities, sexual intercourse, and infections (6). Interestingly, female participants and those with somatic sensory hypersensitivity, are more likely to report various non-dietary factors as triggers than male participants and those without hypersensitivity (6). Female participants (32.1%) reported use of sleeping aids to manage IC/BSP flares (6). Although suggestive of a sleep disruption/disturbance, participants’ use of sleeping pills may be for the anxiolytic properties, consistent with participants reporting stress as a flare trigger (6).

Few studies have addressed flare triggers, underlying mechanisms, management, and treatment options (6, 11–13). The current study describes a novel mouse model where administration of CYP when combined with 7 days of stressor presentation (CYP+RVS) results in increased voiding frequency and increased somatic sensitivity. However, neither the CYP treatment alone or the 7 days of RVS alone increase voiding frequency or somatic sensitivity in C57Bl/6 mice despite increases in inflammatory mediators (e.g., NGF, BDNF, IL-6, CXC) and histological changes in the urinary bladder. We refer to this mouse model as a stress-induced symptom exacerbation (SISE) mouse model. Our longer-term goal is to use this mouse model to evaluate potential targets, management, and treatment interventions when stress is a contributing factor to flares. The SISE model in female mice demonstrate increased voiding frequency and increased pelvic and hindpaw sensitivity. Although increases in both pelvic and hindpaw sensitivity have been demonstrated previously in some animal urinary bladder inflammation models and may reflect overlapping dermatomes of the urinary bladder and hindpaw, not all models of urinary bladder inflammation (e.g., mice with chronic urothelial overexpression of NGF, NGF-OE) (44) exhibit both pelvic and hindpaw sensitivity. It would be of interest in future studies to evaluate upper limb sensitivity as well as other body regions with dermatomes distinct from the urinary bladder.

We also demonstrated increased expression of inflammatory mediators in the urinary bladder including neurotrophic factors (e.g., NGF, BDNF), chemokines (CXC) and IL-6 expression in the urinary bladder consistent with a large body of literature demonstrating roles for inflammatory mediators in preclinical animal models of and in IC/BPS patients (18, 21–34). Urinary bladder neurotrophic factor (i.e., NGF, BDNF), CXCL9, CXCL10 and IL-6 expression was increased in mice with CYP alone, RVS alone and CYP+RVS compared to control mice. In mice treated with CYP alone, the urinary bladders also exhibited physical signs of irritation/inflammation including urothelial erosion, urothelial cell sloughing and petechial hemorrhages. These studies demonstrate that despite no changes in urinary bladder function in mice treated with CYP alone or exposed to RVS (7-day) alone, changes in the urinary bladder inflammatory environment were present. This is consistent with the suggestion that low concentration (35 mg/kg) CYP treatment or RVS (7-day) exposure results in an underlying bladder inflammation that can be exacerbated when CYP and RVS are combined (CYP+RVS) producing changes in bladder function (i.e., increased voiding frequency) and enhanced somatic sensitivity. With the development of this SISE model, we have many follow-up questions that we wish to address including (1): How long do changes in bladder function and somatic sensitivity persist after cessation of CYP+RVS? (2) Can changes in bladder function and somatic sensation be reinstated with CYP or RVS alone? (3) Is 7-day RVS necessary when combined with CYP to elicit changes in bladder function and somatic sensation or can shorter duration RVS produce similar effects?

Multiple animal models of stress (e.g., including social stress (52–55), immobilization stress (56–59), water avoidance stress (60–62), footshock (63–65)) produce morphological, inflammatory and functional changes in the urinary bladder, and visceral sensitivity similar to signs and symptoms of IC/BPS. Among different types of stress, psychosocial stress may be the most relevant to everyday life. Psychosocial stress results from one’s cognitive appraisal of a perceived threat (either real or imagined) and our determination that we may not be able to overcome, adjust or adapt to that challenge (35, 66–71). Classically, the heightened HPA axis coordinates sympathetic nervous system activity and cortisol responses to maintain homeostasis (35, 66–71); however, repeated challenges and adversity or the inability to appropriately attenuate stress responses can result in mal-adaptations and cumulative and persistent damages (i.e., increased HPA and sympathetic reactivity; allostatic overload) that can manifest a variety of disorders including anxiety, depression, and gastrointestinal and urinary tract disorders (35, 36, 66–78).

A repeated variate stress (RVS) paradigm (7-day) in rats has been used to examine neurochemical expression in the bed nucleus of the stria terminalis (15, 17). This model of RVS was found to be anxiogenic, most likely mediated by neural circuits in the BNST (15, 79–82). Among other animal models with daily presentation of the same stressor, the RVS paradigm may be more relevant to human daily life stressors and lacks habituation because of novel stressor exposure. Plasma corticosterone, a steroid hormone involved in the HPA axis secreted by the adrenal gland in rodents during stressor exposure, is significantly increased (5.2-fold) in mice (14) and adrenal gland weights (37) in rats are increased following RVS. We have previously used the RVS protocol to characterize its effects on bladder function and somatic sensitivity in the rat (37, 38) and PACAP promoter-dependent EGFP BAC transgenic mouse strain (PACAP-EGFP) (14). RVS decreased (1) bladder capacity and void volume and increased voiding frequency in rats and PACAP-EGFP mice (14, 37) (2); increased somatic sensitivity of both the hindpaw or pelvic regions in rats and PACAP-EGFP mice (14, 37); and (3) produced changes in the inflammatory milieu (e.g., histamine, myeloperoxidase, NGF, chemokines) of the urinary bladder in rats (37, 38). It should be clarified that a 7-day RVS protocol in rats (15, 17, 82) and in specific transgenic mice (14) produced anxiety-like behaviors and changes in voiding function. However, other ongoing studies from our laboratory (83) and studies from other laboratories (S.E. Hammack, personal communication) indicate that using a 2-week RVS protocol in C57Bl/6 (wildtype) mice is needed to induce consistent anxiety-like behaviors and changes in voiding function.

NGF has been implicated in the peripheral sensitization of nociceptors (84–86), contributes to bladder sensory function and the development of referred hyperalgesia in response to urinary bladder inflammation (22, 44, 85–91). In addition, elevated NGF levels have been detected in the urine and urinary bladders of women with BPS/IC (23, 28). Intravenous administration of a humanized NGF neutralizing monoclonal antibody (tanezumab) in IC/BPS patients demonstrated proof of concept by improving the global response assessment and reducing the urgency episodes (92). BDNF is also upregulated in micturition reflex pathways in both humans and rodents with cystitis (93, 94) because of increased NGF synthesis (95–98). IC/BPS patients report symptom improvement following BDNF reduction (99). Pharmacological blockade of both NGF and BDNF in bladder inflammation models produce complementary improvements in bladder function (21, 100, 101). CYP+RVS mice exhibited significant increases in urinary bladder NGF and BDNF expression. Future studies are addressing NGF and/or BDNF blockade in the SISE model to determine effects on urinary bladder function and somatic sensitivity.

The underlying neural substrates, mechanisms and pathways linking psychosocial stress to altered behaviors and physiological disorders are still unclear. We previously used PACAP-EGFP transgenic mice to map specific neuronal PACAP populations in the CNS following RVS and/or RVS and bladder function testing (102). Bladder function testing in mice exposed to RVS increased numbers of PACAP-EGFP+ cells in lumbosacral spinal cord and dorsal root ganglia and supraspinal locations including the locus coeruleus, Barrington’s nucleus, rostral ventrolateral medulla, PAG, raphe, and amygdala (103). These supraspinal regions have reciprocal connections with the HPA axis which may represent some of the anatomical substrates connecting stress, bladder function and HPA function (104–106). Additional studies are needed to understand how stress or the failure to attenuate stress signaling can result in maladaptation and cumulative long-term damages that can manifest a variety of disorders including urinary bladder dysfunction (35, 66–69, 71, 105, 106). We have previously demonstrated improvements in bladder function following intravesical instillation of the PAC1 receptor antagonist, PACAP6-38, following RVS exposure in mice. Current studies are evaluating the contribution of PACAP and associated receptors in this SISE model.

IC/BPS patients exhibit HPA dysfunction and increased levels of stress (1, 107). Abnormal, or chronic HPA activation is associated with dysregulated responses to stress and inflammation and may result in aberrant sympathetic activation (1, 108, 109) that may contribute to changes in urinary bladder function and somatic sensation. For example, subcutaneous phenylephrine induced somatic sensitivity, increased c-Fos immediate early gene expression in spinal micturition pathways and bladder hyperactivity (110). These changes were reversed by a selective α1A-adrenoceptor antagonist (i.e., silodosin), and absent in TRPV1^-/-^ mice. In future studies, we will evaluate the involvement of sympathetic activation in the current SISE model and voiding dysfunction by determining serum concentration of noradrenaline and the adrenal response.

In summary, these initial results demonstrated that CYP (35 mg/kg) alone or RVS alone creates a change in the inflammatory environment of the urinary bladder and a systemic increase in corticosterone expression but does not result in a change in bladder function or somatic sensitivity until CYP is combined with RVS (CYP+RVS). The SISE model of CYP+RVS will be useful to develop hypotheses that address the underlying mechanisms that contribute to stress exacerbation of signs and symptoms of functional bladder disorders leading to identification of novel targets, management, and potential treatments.

## Data availability statement

The original contributions presented in the study are included in the article/supplementary material, further inquiries can be directed to the corresponding author/s.

## Ethics statement

The animal study was reviewed and approved by University of Vermont IACUC.

## Author contributions

MV conceived and designed research. SC, BG, and MV performed experiments. SC, BG, and MV analyzed data. BG and MV interpreted results of experiments. MV and BG prepared figures and drafted the manuscript. BG, SC, and MV edited and revised manuscript. All authors contributed to the article and approved the submitted version.
